# Performance evaluation of the next generation solid-state digital photon counting PET/CT system

**DOI:** 10.1186/s13550-018-0448-7

**Published:** 2018-11-06

**Authors:** Jun Zhang, Piotr Maniawski, Michael V. Knopp

**Affiliations:** 1 Wright Center of Innovation in Biomedical Imaging, Department of Radiology, The Ohio State University Wexner Medical Center, 395 W. 12th Avenue, Room 430, Columbus, OH 43210 USA; 2 Philips Healthcare, Cleveland, OH USA

**Keywords:** Digital photon counting, Solid state, Silicon photomultiplier, Positron emission tomography, System performance

## Abstract

**Background:**

The first solid-state silicon photomultiplier (SiPM) digital photon counting (DPC) clinical PET/CT system was introduced by Philips in recent years. The system differs from other SiPM-based PET/CT systems and uses lutetiumyttrium oxyorthosilicate (LYSO) scintillators directly coupled with their own individual SiPM DPC detectors eliminating the need for Anger-logic positioning decoding. We evaluated the system performance, characteristics, and stability of the next generation DPC clinical PET/CT based on NEMA NU2-2012 tests, NEMA NU2-2018 test (timing resolution) and human studies.

**Results:**

An energy resolution of 11.2% was measured. NEMA NU2-2012 tests revealed a spatial resolution (mm in FWHM) from (3.96, 4.01, 4.01) at 1 cm to (5.81, 5.83, 4.95) at 20 cm for (axial, radial, tangential). A 5.7 cps/kBq system sensitivity was measured. Peak noise equivalent count rate (NECR) and peak true count rate could not be determined as each exhibited increasing values up to the maximum activity measured (~ 1100 MBq). The maximum NECR was 171 kcps @ 50.5 kBq/mL, with corresponding scatter fraction of 30.8% and maximum trues of 681 kcps. NEMA hot sphere contrast ranged from 62% (10 mm) to 88% (22 mm), cold sphere contrast of 86% (28 mm) and 89% (37 mm). A timing resolution of 322 ps (^22^Na point source based) and 332 ps (NEMA NU2-2018) was obtained. It revealed < 1% change in TOF timing and ± 0.4% change in energy resolution during 31-month stability monitoring. CQIE assessment found < 3% axial variance in SUV. 100–60% recovery coefficients of activity concentration at various sphere sizes and contrast levels were measured.

**Conclusions:**

This scanner represents the first solid-state DPC PET/CT, a technologic leap beyond photomultipliers tubes and anger logic. It presents considerable improvements in system performance and characteristics with excellent time-of-flight capability compared to conventional photomultiplier tube (PMT) PET/CT systems. The DPC system leads to promising clinical opportunities with excellent image quality, lesion detectability, and diagnostic confidence.

## Background

PET/CT has evolved to be an essential imaging modality in oncology and neuroscience. Up to now, PET detectors have been mainly based on photomultiplier tubes (PMT) which have advantages of high gain signal amplification, multichannel capabilities and good timing performance, however have also disadvantages of limited photon-to-electron quantum conversion efficiency and relative bulkiness [1, 2]. Recent efforts pushed beyond these limitations to further improve PMT PET/CT system performance [3–5].

Modern solid-state detectors were developed in the 90s using silicon metal oxide semiconductor (MOS) structures with avalanche breakdown mode operation capable of detecting single light photons [6]. An initial concept of Geiger-mode avalanche photodiodes (APDs) or silicon photomultipliers (SiPMs) was proposed in the late 90s combining with many features of PMTs and traditional APDs [7]. Analog SiPM detectors started to be available for medical applications around 2004 [8] and were introduced to more imaging applications with relative compact design and better MR compatibility however initially without time of flight (TOF) capabilities [9–16]. Analog SiPM detectors using light-sharing designs use single photon avalanche diode (SPAD) arrays to detect single scintillation photons, while the pulses generated by multiple SPADs are combined into one analog output signal requiring off-chip processing and anger logic decoding [17].

Philips Healthcare introduced the first solid-state, SiPM digital photon counting (DPC) PET/CT system at RSNA 2013. Before that, its prototype system demonstrated better image quality and diagnostic accuracy compared with Gemini TF PET [18]. The DPC detector differs from analog SiPM detectors: each SPAD is part of an array running as a digital counter, and the readout of the digital signal provides a direct count of the number of scintillation photons detected without analog signal processing [17, 19]. The system uses lutetiumyttrium oxyorthosilicate (LYSO) scintillators directly coupled with individual SiPM DPC detectors eliminating the need for Anger-logic positioning decoding [20, 21].

This medical physics focused study evaluates the DPC system performance characteristics in sensitivity, noise equivalent counting rate (NECR), spatial resolution, image quality, energy resolution, and TOF timing resolution using National Electrical Manufacturers Association (NEMA) NU2-2012 and the recent NU2-2018 as well as other methodologies.

## Methods

### System specifications

**Fig. 1:**
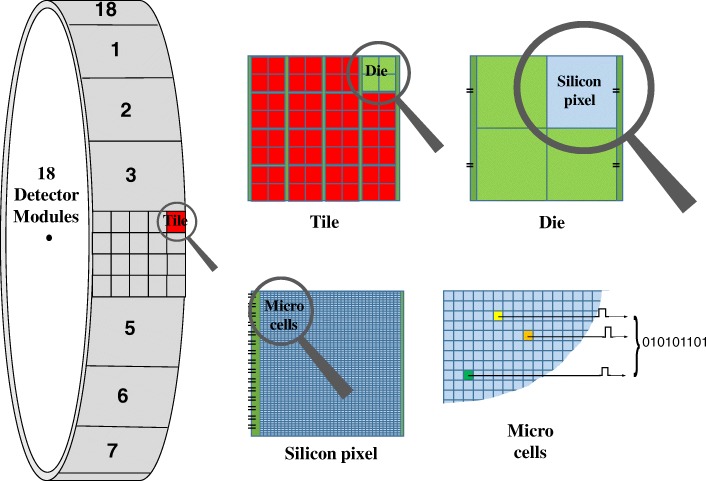
Solid-state digital photon counting PET detector design. When a scintillation photon hits the microcell sensor, the integrated photon counter increases and the integrated timer measures the arrival time of the individual photon on that die. The chip measures all photons during the desired duration of the detection process. The values of the integrated photon counter and timer are read out via a digital interface

The Vereos PET/CT (dPET) (Philips, Cleveland OH) is a system based on SiPM DPC technology coupled with a 64-slice helical CT of the Ingenuity class, and its first system was operational at Wright Center of Innovation in Biomedical Imaging (WCIBMI) at The Ohio State University (OSU) since 07/2014. This system fulfilled all system specifications of the commercially released system (10/2017) and was used to perform clinical trials for regulatory submissions. The CT uses a 40 mm axial field of view (FOV) with 3D dose modulation for low dose CT capabilities integrated with an iterative reconstruction technique (iDose^4^) and the Metal Artifact Reduction for Orthopedic Implants algorithm. The 764 mm PET detector ring spans 164 mm in the axial FOV and is composed of 18 flat detector modules with 4 by 5 array SiPM detector tiles on each module. Every tile consists of a 4 × 4 matrix of sensor silicon dies, and each die is a 2 × 2 matrix of digital photon counter detectors (silicon pixels). Every pixel couples directly to a 3.86 × 3.86 × 19 mm^3^ single LYSO crystal for a total of 23,040 crystal-DPC pairs. 3200 SPAD (microcells) are integrated on each pixel resulting in 73,728,000 microcells that enable detection of single scintillation photons with each photon being converted directly into a pure binary output signal (Fig. 1). The number of microcells recording a photon is read out as the total number of photons detected by a pixel.

PET data are acquired in 3D mode and stored in a list-mode file containing position, timing, and energy information of each DPC-collected event. PET reconstruction is performed using a list-mode-based TOF OSEM algorithm with blobs as basic functions [22, 23]. It uses a Monte Carlo-based scatter simulation for scatter correction [24] and Casey averaging for smoothed randoms estimation [25]. The system applies 2.0 ns, 4.0 ns, and 4.6 ns coincidence windows depending on the transaxial FOVs (256 mm for brain, 576 mm and 676 mm for body). PET reconstruction uses isotropic voxels for standard definition (SD) (4 × 4 × 4 mm^3^), high definition (HD) (2 × 2 × 2 mm^3^) and ultra-high definition (UHD) (1 × 1 × 1 mm^3^). Post-reconstruction image processing includes an optional Richardson–Lucy, maximum-likelihood resolution recovery implementation and Gaussian filtering [26–28].

### Digital photon counting (DPC)

**Fig. 2:**
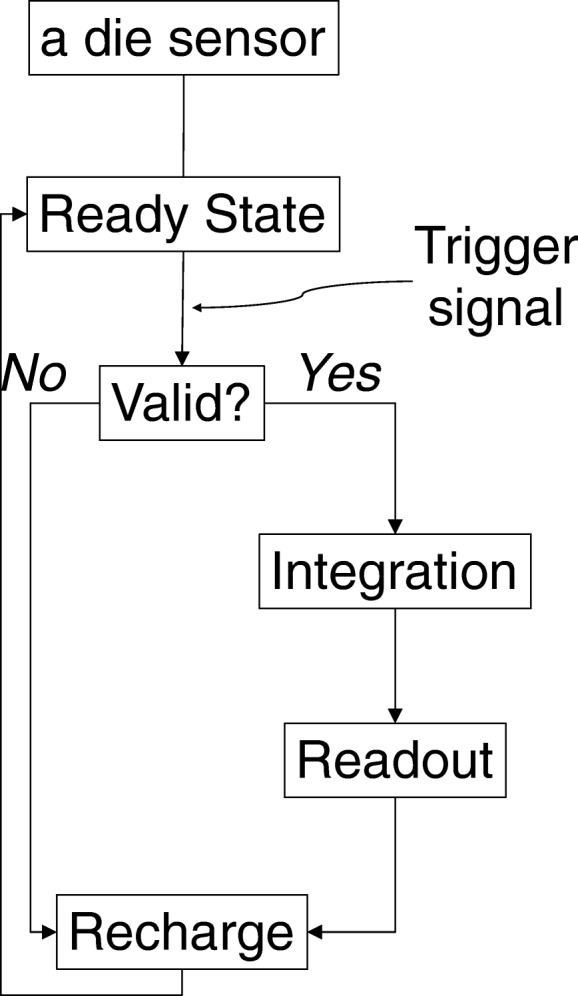
Single event-based data acquisition sequence on each die sensor of the investigated DPC PET system

**Fig. 3:**
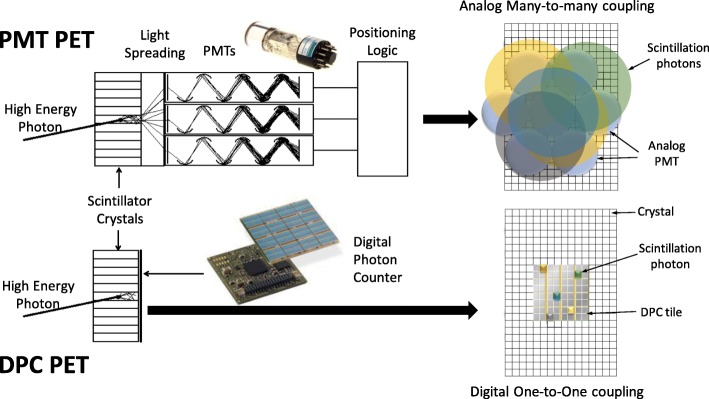
Comparison of PMT PET detector and DPC PET detector approach. For PMT PET, incident photons are converted to visible light via interaction with scintillators coupled with multiple PMTs to generate and multiply electronic signals for further Anger-logic positioning decoding which exhibits significant dead time. For DPC PET, 1:1 coupling of scintillator and digital detector directly channels lights from a crystal to its ‘own’ detector with a digital signal output that is virtually dead time free

The DPC technology allows every microcell to be individually activated or inactivated, SPADs and data processing are integrated onto a single silicon chip at each pixel allowing fast and accurate ultra-low light single photon detection, and each microcell directly detects and counts the breakdown of an individual SPAD on its silicon chip. A light photon is detected by one of the integrated 3200 microcells yielding a pure binary signal measured by the on-chip photon counter and timer. Background noise, or say dark count rate (DCR), is measured and managed effectively for each individual counter. The biggest contribution to the overall sensor DCR is caused by only a small percentage of the cells (such as ~ 10% cells responsible for 70–80% DCR). Disabling high dark count rate cells on a small active sensor area leads to significantly reduced overall sensor DCR; therefore, the overall DCR can be greatly reduced by switching off the noisiest cells. Each sensor operates independently from the other sensors. Only those die sensors that detect sufficient photons reaching the configured thresholds will start the acquisition sequence (Fig. 2). In the beginning, a die sensor is in the “ready state,” where all its microcells are fully charged, and the system waits for the start of the photon events until a trigger occurs. When the number of photons detected in a silicon pixel becomes higher than the configured threshold, it prompts a timestamp to be saved and begins a validation process to detect a user-configured number of further photons within a certain time. If this validation threshold is exceeded, there is a subsequent integration period before a readout process sends data (four photon count values, one per silicon pixel on the die, and one timestamp per event) to a readout buffer. After readout, the microcells are recharged so that the die is ready for further data acquisition. If the validation threshold was never reached, all microcells are immediately recharged and go back to the “ready state.” At the end, the photon counter is read out by summing overall detected photons with a timestamp, and the whole process is entirely digital without need of signal amplification. The localization determination of the scintillation light created by gamma-ray interaction using the DPC compared to traditional PMT-based approach is shown in Fig. 3.

### System performance measurements

System performance measurements of the dPET utilized NEMA NU 2-2012 procedures [29] for spatial resolution, sensitivity, scatter fraction, count loss, NECR, and image quality.

Spatial resolution of a PET scanner is an intrinsic feature of the device, which reflects the ability of the system to distinguish between two points after image reconstruction, and is usually measured in transaxial (radially and tangentially) and axial directions. Spatial resolution of the system was measured using a point source of 4 MBq ^18^F in a capillary tube (tube inside diameter ≤ 1 mm, tube length = 100 mm, axial extend of the activity: approximately 1 mm). Measurements were performed at the center of axial FOV and three-eighths of the axial FOV from the center of the FOV with (*x*, *y*) locations of (0, 1), (0, 10), and (0, 20) cm. For the axial resolution measurements, we rotated the capillary with 90° which was perpendicular to the long axis of the system so that the axial extent of the source was within the diameter of the capillary (1 mm or less). List-mode data were reconstructed using 3D Fourier re-projection with an unapodized filter [30]. Pixel size of the reconstructed images used for spatial resolution calculation was 1 × 1 × 1 mm^3^. Axial, radial, and tangential resolutions in full width at half-maximum (FWHM) and full width at tenth-maximum (FWTM) for each radius (1, 10, and 20 cm), averaged over both axial positions were calculated and reported.

Sensitivity of PET represents the ability to detect true coincidence events in cps/kBq, which typically depends on solid angle, system photon detection efficiency, dead time and other factors, and is measured based on established techniques [31, 32]. NEMA sensitivity was measured at the center and 10 cm off in transverse FOV using five aluminum sleeves (Data Spectrum Corp., Hillsborough, NC; 70 cm in length, inner diameters 3.9–16.6 mm) and a length of 70 cm plastic tubing with 6 MBq ^18^F. The initial activity was calibrated in a dose calibrator which in general has less than 1% accuracy error. Successive measurements were performed 10 min post ^18^F injection by adding aluminum sleeves of known attenuation. Decay-corrected count rate was summed for all slices to give the total count rate for each sleeve and then extrapolated to an attenuation free measurement.

Measurements of scatter fraction, count losses, and NECR were performed using the NEMA PET scatter phantom. A plastic tubing (3.2 mm inner diameter) filled with ~ 1100 MBq ^18^F over a length of 70 cm was measured over 16-h until the activity decayed to a low level with true events losses of < 1%. Data were binned into sinograms and used to calculate the scatter fraction and count rates.

For measurements of image quality and scatter corrections, a NEMA IEC Body phantom with ^18^F (75.9 MBq) was abutted with a 70 cm scatter cylinder phantom containing ^18^F line source (140 MBq) to approximate the clinical situation of having activity extending beyond the scanner. The imaging phantom contains two cold spheres (28 and 37 mm) and four hot spheres (10–22 mm), positioned circular with a radius of 57.2 mm at the center of the phantom similar to NEMA NU 2-2012 standard. The sphere-to-background ratio (SBR) was 4. Acquisition was repeated three times with 180 s/bed. Contrast for each hot sphere (*Q*_*H*_) and each cold sphere (*Q*_*C*_) was calculated as

$$ {Q}_H=100\%\times \frac{C_{M,S}/{C}_{M,B}-1}{C_{T,S}/{C}_{T,B}-1} $$ and $$ {Q}_C=100\%\times \left(1-\frac{C_{M,S}}{C_{M,B}}\right). $$

where *C*_*M,S*_ and *C*_*M,B*_ are the averaged activity concentration in a circular ROI with a diameter equal to the inner diameter of the sphere (*S*) being measured (*M*) or in the background (*B*) and *C*_*T,S*_*, C*_*T,B*_ are the true (*T*) activity concentration.

The lung error was calculated by averaging the relative lung errors of each slice in axial direction according to the NEMA NU2-2012 definition.

Energy and timing resolution are not part of the standard NEMA NU2-2012 testing. The coincidence timing resolution as a function of activities was measured using the NEMA NEC phantom data according to NEMA NU2-2018 standard and its associated methodologies [33–35]. For the stability monitoring, energy and timing resolution were additionally measured using built-in PET daily quality check (QC) procedures. Energy window was set at 88–120% of 511 keV (450–613 keV), and the coincidence-timing window was set at 4 ns with a delayed coincidence window technique utilized to estimate the random coincidences. A 3.7 MBq ^22^Na point source centered in PET FOV was used with daily acquisition of PET coincidence (~ 200 Mcounts). Energy and timing resolution were then calculated separately from event histograms with 4 keV bins for energy and 19.6 ps bins for timing. In addition, sensitivity as a function of activities was separately measured using the NEMA NEC phantom data [34, 35].

### System stability

System stability of sensitivity (in relative count rate), energy resolution, and timing resolution were monitored over a 31-month period between October 2014 and May 2017. These parameters were extracted from the built-in PET daily QC and corresponding system log files.

### PET image quality assessment

PET image quality was additionally evaluated for uniformity, partial volume effect (PVE), maximum recovery coefficients (RC_max_), and clinical lesion detectability.

PET uniformity assessment was performed according to the NCI Center for Quantitative Imaging Excellence (CQIE) program guidelines [36]. A 9293 mL water cylinder phantom with 53 MBq FDG was scanned and reconstructed using 3D OSEM TOF. A 2D circular region-of-interest (ROI, ~ 200 cm^2^) was placed across all slices. Images were analyzed according to CQIE criteria (SUVmean of 0.9–1.1 with ≤ 10% variances in axial).

For PVE assessment, a Jaszczak Flangeless Esser PET phantom with rod inserts (4.8–12.7 mm) was imaged at 60 min post 48 MBq ^18^F-FDG injection with 90 s/bed for body and 10 min for brain according to ACR accreditation guidelines [37]. Images were reconstructed in SD, HD, and UHD with different voxel sizes.

The phantom procedures different from the NEMA NU 2-2012 standard are detailed below for assessment of RC: a NEMA IEC Body phantom (185 ± 3.7 MBq ^18^F-FDG) filled with 6 hot spheres (10–37 mm) and varying SBRs of 2, 4, 8, 16, 32, 64, and 100 was imaged with 75 s/bed and reconstructed in SD (4 × 4 × 4 mm^3^) using 3D OSEM TOF (3i15s for dPET and 3i33s for cPET). Spherical ROIs with approximately 37 mm in diameter were placed on each of the spheres. RC_max_ was analyzed and defined as

$$ {\mathrm{RC}}_{\mathrm{max}}=\frac{C_{M,S}}{C_{T,S}} $$

where *C*_*M,S*_ is the maximum of the measured (*M*) activity concentration in each sphere, and *C*_*T,S*_ is the true (*T*) activity concentration for sphere ROIs.

Assessments of clinical image quality and lesion detectability were performed based on PET/CT scans of clinical patients. PET/CT scans were acquired with 90 s/bed on the dPET before or after local standard of care PET/CT on a conventional PMT-based Gemini TF 64 system (cPET, 90 s/bed, 75 min uptake, 481 MBq ^18^F-FDG). All data were reconstructed using 3D TOF OSEM with 3 iterations (subset of 15 for SD dPET, and 33 for SD cPET). dPET CT was reconstructed using iDose^4^ in 4 mm and used for PET attenuation correction. SUV_peak_ values of representative lesions were calculated using MIMSoftware 6.7 (MIM Software Inc.). The study (NCT02283125) was approved by the institutional review board, and all subjects signed an informed consent.

## Results

### System performance measurements and system stability

NEMA spatial resolution, sensitivity, NECR, count rate, scatter fraction, image quality, and timing resolution as well as the system stability in timing resolution, energy resolution and sensitivity are presented.

Table 1 demonstrates the spatial resolution of the dPET system, ranging from (in FWHM) 3.96 mm (axial), 4.01 mm (radial), and 4.01 (tangential) at the center toward the periphery of 5.81 mm (axial), 5.83 mm (radial), and 4.95 mm (tangential) at 20 cm off center.

**Table 1:** NEMA spatial resolution of the dPET system

Spatial resolution												
	Measurements 01^a^						Measurements 02					
	FWHM			FWTM			FWHM			FWTM		
Direction	1 cm	10 cm	20 cm	1 cm	10 cm	20 cm	1 cm	10 cm	20 cm	1 cm	10 cm	20 cm
Axial	3.96	4.83	NA	8.37	9.45	*NA*	4.14	4.72	5.81	8.51	9.30	11.76
Transverse (radial)	4.11	4.47	NA	8.38	8.72	*NA*	4.01	4.64	5.83	8.37	8.97	10.41
Transverse (tangential)	4.11	4.38	NA	8.38	8.94	*NA*	4.01	4.41	4.95	8.37	8.91	10.43

Spatial resolution is measured in millimeters with radial offset at 1 cm, 10 cm, and 20 cm

^a^Measurement 01 contains all tests except for the radial offset at 20 cm; we repeated all test including the 20 cm offset results as shown in measurements 02

**Fig. 4:**
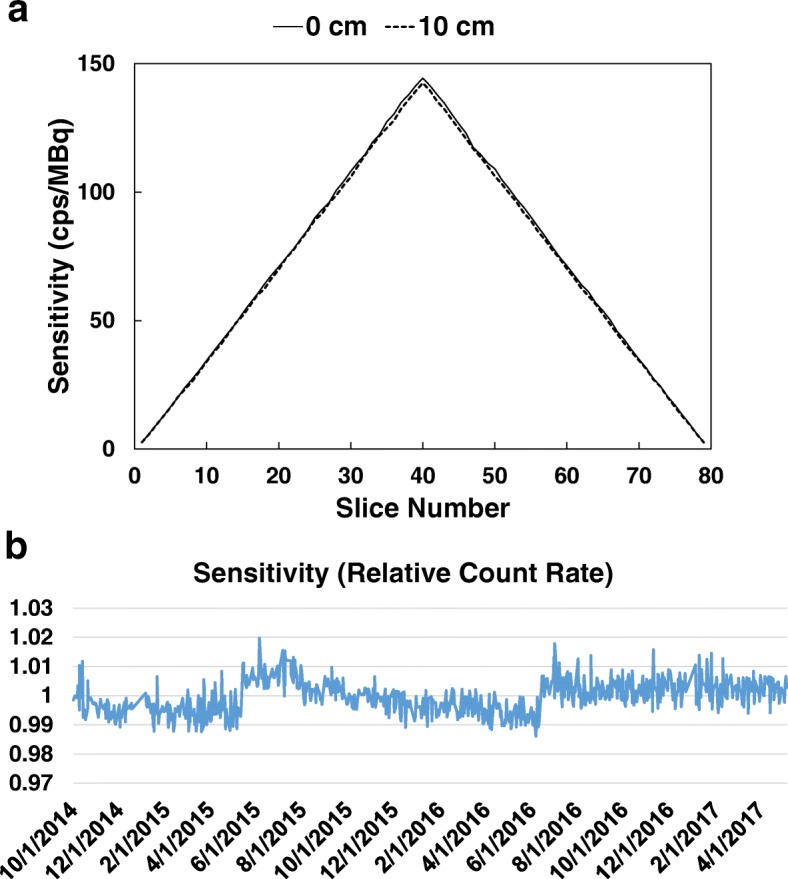
NEMA sensitivity of the dPET system with (**a**) NEMA sensitivity profile as a function of axial slices and (**b**) sensitivity stability over 31 month (< 1.5% variability) with two jump points in June 2015 and July 2016 after system recalibration

The average NEMA sensitivity of the dPET system was 5721 cps/MBq at the center and 5637 cps/MBq at a 10 cm radial offset from the FOV center. Figure 4a details the measured NEMA sensitivity profile along axial slices, and Fig. 4b shows the stability of system sensitivity over 31 months (better than 1.5% variability) by monitoring the relative count rate as a surrogate for sensitivity compared to baseline.

**Fig. 5:**
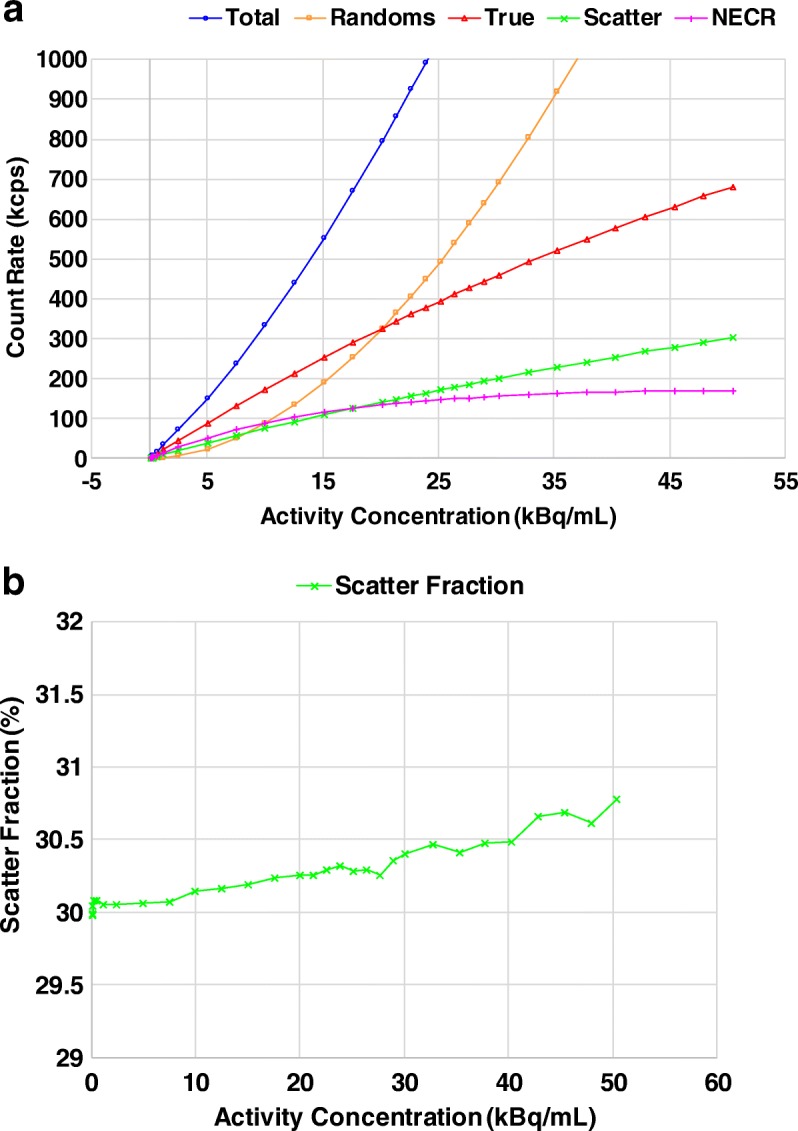
NEMA count rate measurements as a function of activity concentration. **a** Count rate of total, randoms, true, and NECR. **b** Scatter fraction

Figure 5 summarizes count loss measurements of the dPET as a function of activity concentration. With the amount of ~ 1100 MBq (~ 30 mCi) ^18^F used for the count loss test, neither the peak true count rate nor the peak NECR could not be found as the system did not show a peak value beyond which the NECR began to decrease with increasing activity. Consequently, in this study, we use ‘maximum’ instead of ‘peak’ to describe these results. It revealed a maximum NECR of 171 kcps at 50.5 kBq/mL, with corresponding true count rate of 681 kcps and scatter fraction of 30.8% at the maximum NECRs, respectively.

**Fig. 6:**
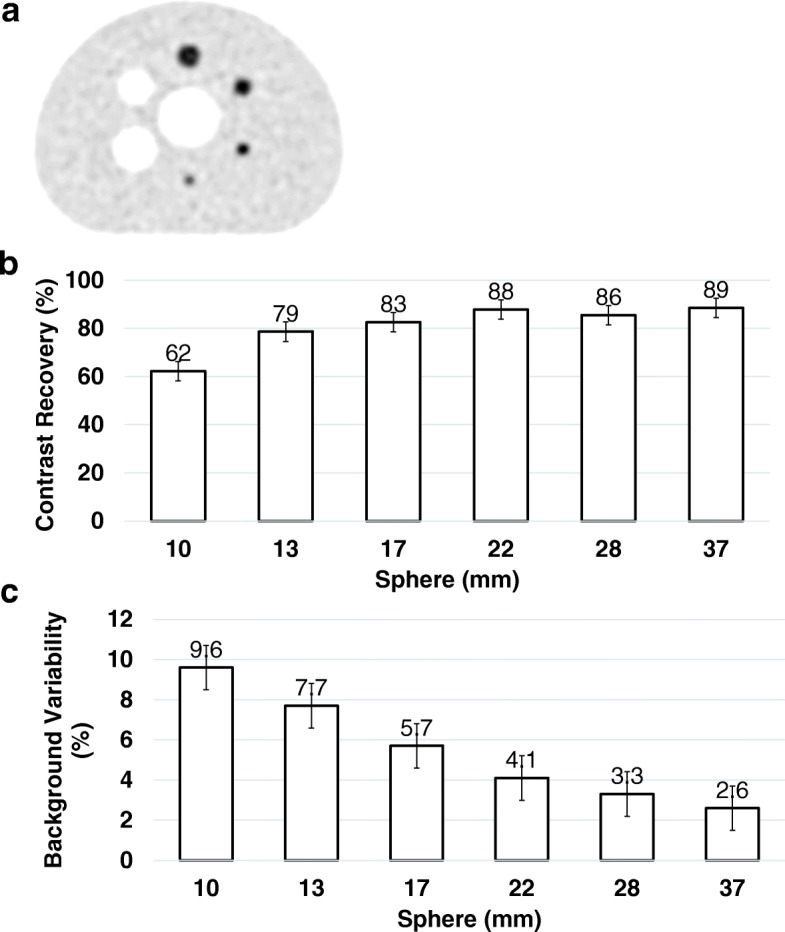
NEMA NU2-2012 image quality of the IEC body phantom PET reconstructed using 3D OSEM TOF PSF. **a** PET image (**b** and **c**), assessment of contrast recovery, and background variability, respectively. The average lung error was 4.18 ± 0.05%

NEMA image quality of the IEC body phantom PET reconstructed in HD using 3D OSEM with point spread function (PSF) is shown in Fig. 6a. Image contrast recovery and background variability are given in Fig. 6b and c. The average lung error was 4.18 ± 0.05%.

**Fig. 7:**
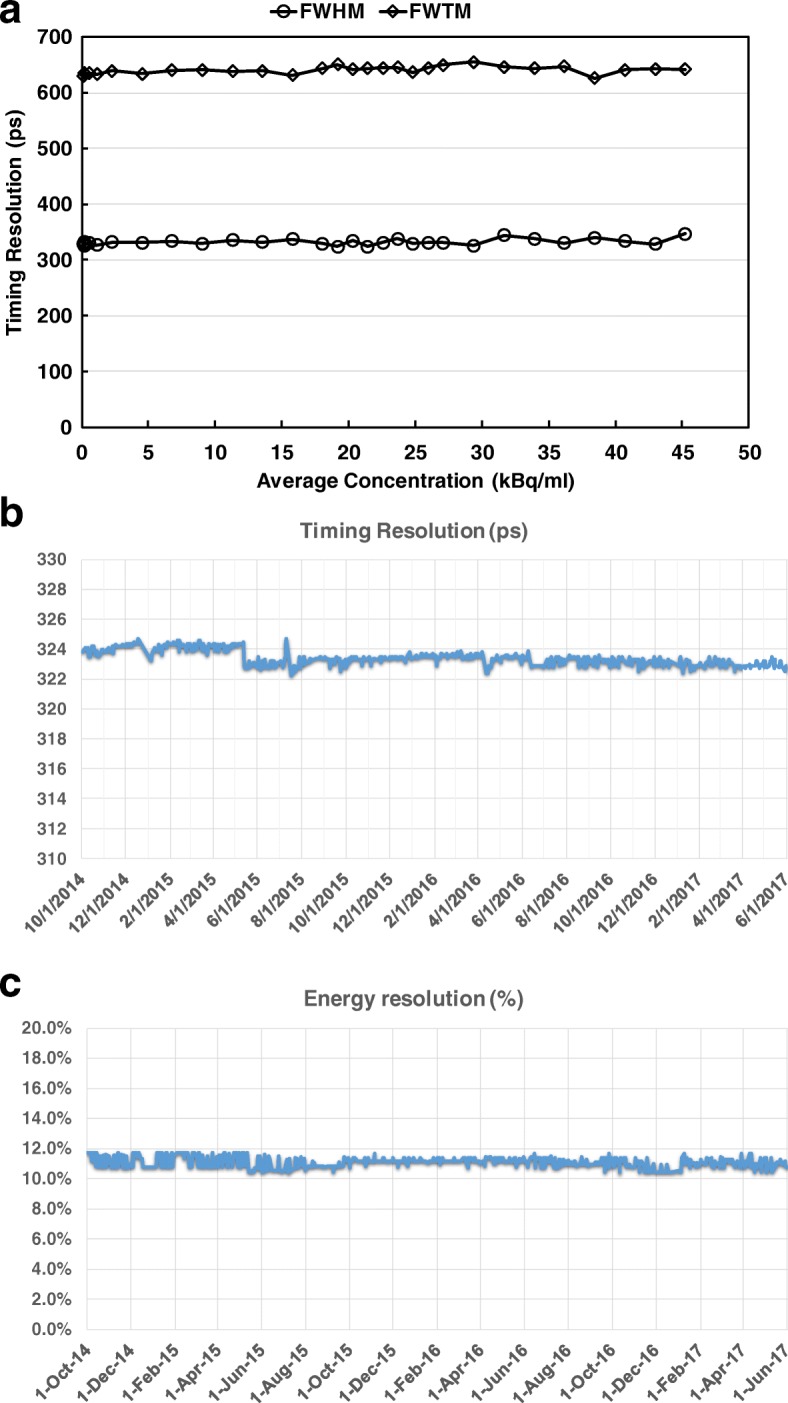
Timing resolution and energy resolution of the dPET system. **a** NEMA NU2-2018 timing resolution in FWHM and FWTM as a function of activity concentration. **b** Stability of timing resolution (^22^Na point source based) within the 31-month monitoring time window. It demonstrates a slight improvement from 325 ps to 322 ps when system calibration was performed 6 months after installation. **c** Stability of energy resolution during the monitoring time period

Results of timing and energy resolution of the dPET system are shown in Fig. 7. Figure 7a demonstrates the NEMA NU2-2018 coincidence timing resolution as a function of activity concentration which was measured as 332 ps in FWHM and 640 ps in FWTM, with robust distribution (1 ± 2% for FWHM and 2 ± 1% for FWTM) obtained as increasing activity concentration from 0 to 50.5 kBq/mL. Fig. 7b and c shows the stability of timing and energy resolution (^22^Na point source based) within the monitoring time window. It revealed < 1% variability for timing and < 0.5% variance for energy. The average energy resolution was about 11.2% FWHM.

### Additional phantom measurements

**Fig. 8:**
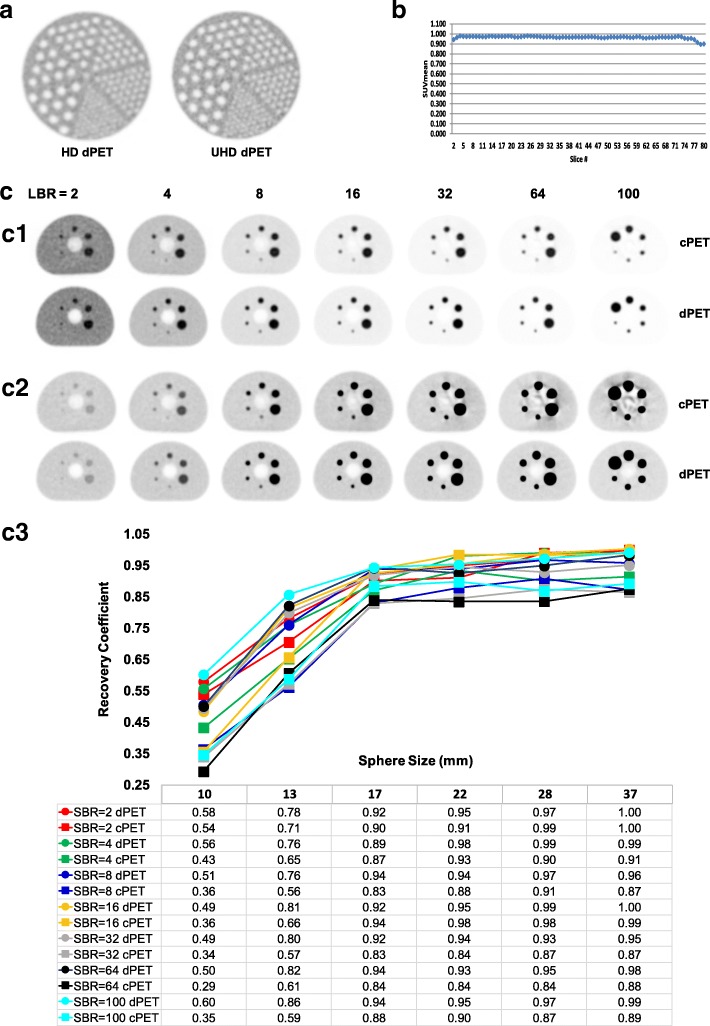
Image quality of dPET via various phantom measurements. **a** HD and UHD dPET images of the ACR phantom cold rods. **b** CQIE dPET uniformity. **c** Comparison of dPET vs cPET of the NEMA IEC Body phantom images at different SBRs: (c1) window-level setting: SUV LL = 0, UL = SUV_max_ of cPET at each SBR; (c2) window-level setting: same background with SUV LL = 0, UL = 5.0; (c3) RCs of dPET vs cPET as a function of sphere sizes (10–37 mm) at different SBRs (2–100)

Figure 8 summarizes dPET image quality using different phantom measurements. dPET images of the ACR phantom is shown in Fig. 8a. With the reduced PVE using smaller voxels, the smallest 4.8 mm rods are detectable on both HD and UHD dPET. Fig. 8b shows the CQIE uniformity of dPET (256 mm FOV) giving < 3% axial variation in SUV which is well below the CQIE 10% acceptable threshold. Images of the IEC body phantom varying with SBRs are compared between dPET and cPET shown in Fig. 8c. Increasing SBR to 16 and above, deformation of the spheres and artifacts for the lung insert appeared to be more and more visible on cPET but not dPET images, and better sphere delineation with enhanced contrast adapting to different SBRs was found on dPET (the potential reason is given in the “Discussion” section). Quantitatively, it indicated an average differences of (− 7 ± 4%, − 6 ± 5%, − 5 ± 5%, − 6 ± 5%, − 22 ± 8%, and − 28 ± 12%) for spheres of (37, 28, 22, 17, 13, and 10 mm) over all SBRs, and an average differences of (− 3 ± 4%, − 10 ± 7%, − 14 ± 10%, − 7 ± 12%, − 16 ± 11%, − 19 ± 13%, and − 18 ± 15%) for SBRs of (2, 4, 8, 16, 32, 64, and 100) over all spheres, for RC_max_ of cPET compared to dPET.

### Clinical patient studies

**Fig. 9:**
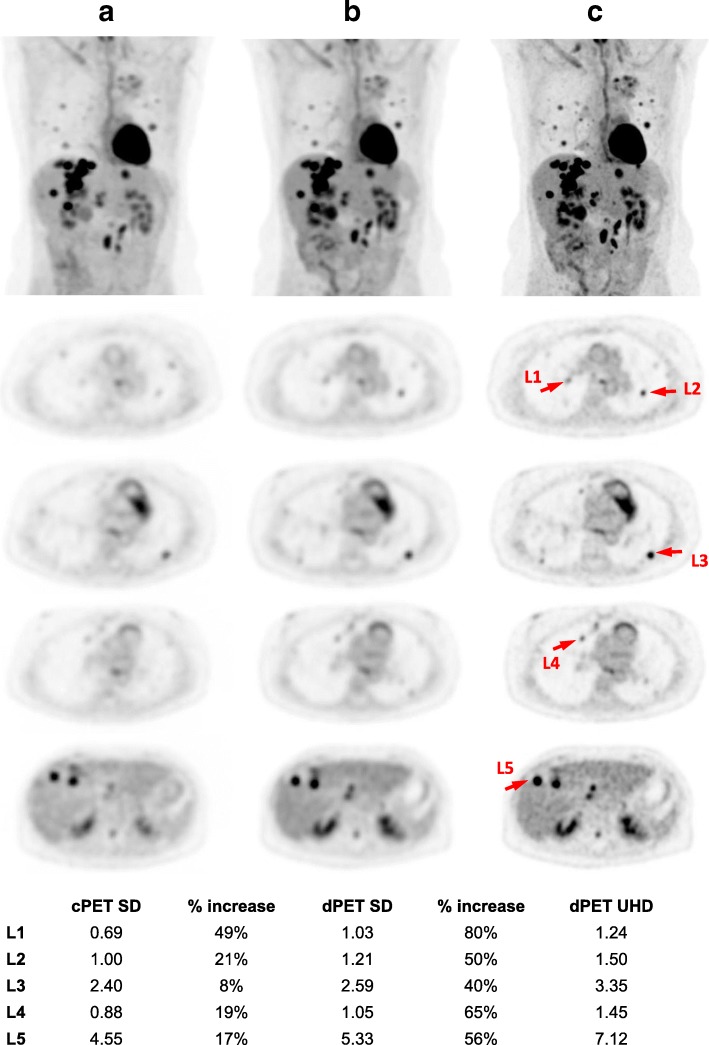
Intra-individual comparison of **a** SD cPET (75 min p.i., 3i33s) with **b** SD dPET (103 min p.i., 3i15s) and **c** UHD dPET (103 min p.i., 3i13s) for a clinical patient with extensive metastatic disease in the lung and the liver (BMI = 18.5, 503 MBq FDG, 90 s/bed). Images were reconstructed using 3D OSEM TOF. The contrast of lesions is improved on SD dPET and prominently on UHD dPET compared to SD cPET. The lesion conspicuity and detectability as well as the diagnostic confidence for the pulmonary and hepatic lesions are classified by blinded reader analysis as substantially improved on dPET. It revealed an average of 23 ± 16% and 58 ± 15% higher SUV_peak_ values for SD dPET and UHD dPET than SD cPET based on five representative lesions selected (pointed by red arrows), respectively

While patient studies are not the focus of this paper, Fig. 9 presents an intra-individual comparison between cPET and dPET of the same clinical patient, which demonstrates improved lesion detectability with better contrast of lesions on the 322 ps dPET compared to the 550 ps cPET.

**Fig. 10:**
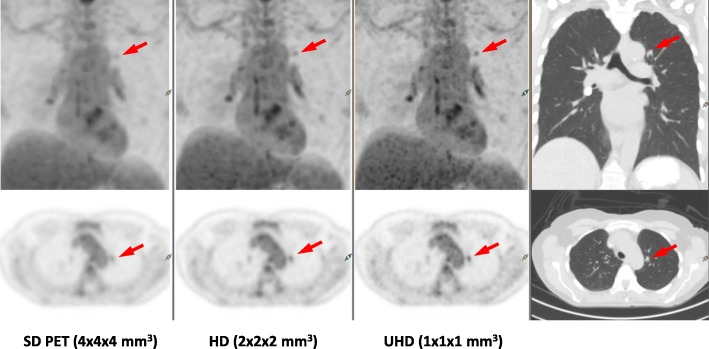
Anal cancer patient scanned on the dPET system (BMI = 24, 481 MBq FDG, 50 min uptake time) and reconstructed in SD, HD, and UHD using 3D OSEM TOF algorithm, in contrast with CT image on the right. The small lesion (red arrows) becomes more visible from SD PET to UHD PET with less PVE noted in the smaller isotropic voxel images

Figure 10 presents a clinical FDG case scanned on the dPET system and reconstructed using SD, HD, and UHD algorithms by isotropically reducing voxel sizes in consistent to the EARL effort using the DPC system [38]. Dramatically enhanced lesion visibility with improved diagnostic confidence using blinded reader studies [39] was found on HD (8 mm^3^ voxel) and particularly UHD (1 mm^3^ voxel) dPET images.

## Discussion

dPET enables every digital photon counter detector to be directly coupled to a single scintillator and leads to higher count rate detection efficiency across a more uniform scintillator distribution compared to cPET. The individual DPC detector controlling greatly reduces the impact of count rate dependent pile-up on energy and timing resolution, and provides highly stable performance across a large dynamic range of count rates.

The major improvement of the dPET system is its significantly improved TOF timing resolution. The benefit of TOF PET has been well-established and became a benchmark technology [40, 41]. Current cPET has timing resolution of 400–600 ps with a scintillator length range of 12–25 mm [42–50]. With the introduction of SiPM-based photo detection technologies, the available solid-state PET systems are achieving timing resolution in the range of 300–400 ps [21, 51], with the investigated dPET demonstrating 322 ps (Table 2). The 322 ps TOF approximately halves the uncertainty from 600 ps TOF PET systems, limits the noise contribution from fewer voxels during PET reconstruction, improves image SNR, and converges faster in 3D-OSEM TOF reconstructions necessitating less iterative steps to achieve optimal image quality. In addition, the study used both the ^22^Na point source (daily PET QC) and the NEMA NEC phantom data (NEMA NU2-2018 new standard) to determine the coincidence timing resolution. We found that the timing resolution measured using the point source based method is ~ 10 ps better than using the NEMA NEC phantom based methodology. To the best of our knowledge, this is potentially due to the vendor-specific software improvement in daily QC for the timing resolution measurement, as well as the fact that the ^18^F line source in the NEMA NEC phantom is typically not perfectly straight.

**Table 2:** Comparison of system characteristics across manufacturer PET/CT systems

Manufacture	GE	GE	Philips	Philips	Philips	Siemens	Siemens	Toshiba
PET/CT model	Discovery MI (4-ring) [51]	Discovery 690 [43]	Vereos (this work)	Ingenuity TF [44]	Gemini T [42]	Biograph mCT flow [45]	Biograph mCT [46, 47]	Celesteion [48–50]
Photo detector	SiPM	PMT	SiPM	PMT	PMT	PMT	PMT	PMT
Number of detectors	9792	256	23,040	420	560	768	768	480
Scintillator	LYSO	LYSO	LYSO	LYSO	LYSO	LSO	LSO	LYSO
Number of crystals	19,584	13,824	23,040	28,336	28,336	32,448	32,448	30,720
Crystal size (mm^3^)	3.95 × 5.3 × 25	4.2 × 6.3 × 25	3.86 × 3.86 × 19	4 × 4 × 22	4 × 4 × 22	4 × 4 × 20	4 × 4 × 20	4 × 4 × 12
Ring diameter (cm)	74.4	81.0	76.4	90.0	90.3	84.2	84.2	88.0
Axial FOV (cm)	20.0	15.7	16.4	18.0	18.0	22.1	22.1	19.6
Plane spacing (mm)	n/a	n/a	1, 2, or 4	2 or 4	2 or 4	2	2	2
TOF Timing resolution (ps)	375	544	322	502	585	555	527	410
Sensitivity (cps/kBq)	13.7	7.4	5.7	7.3	6.6	9.6	9.7	4.0
Transverse resolution @ 1 cm (mm)	4.1	4.7	4.0	4.8	4.8	4.3	4.4	5.1
Transverse resolution @ 10 cm (mm)	5.0	5.1	4.4	5.1	5.2	4.9	4.9	5.1
Axial resolution @ 1 cm (mm)	4.5	4.7	4.0	4.7	4.8	4.3	4.4	5.0
Axial resolution @ 10 cm (mm)	6.0	5.6	4.8	5.2	4.8	5.9	5.7	5.4
Peak NECR (kcps @ kBq/mL)	193.4 @ 21.9	139.1 @ 29.0	171 @ 50.5	124.1 @ 20.3	125 @ 17.4	185 @ 29	156 @ 31.1	≥51 @ n/a
Energy resolution (%)	9.4	12.4	11.2	11.1	11.5	n/a	11.5	11.3
Scatter fraction at peak NECR (%)	40.6	37	30.8	36.7	27	33.4	32.7	42.7

Improvement of PET spatial resolution is another important feature of the dPET system. The fundamental limits of PET spatial resolution are impacted predominantly by crystal size in addition to positron range, non-collinearity, positioning decoding, and reconstruction methods. These factors limit the effective spatial resolution to different degrees. The spatial resolution can be parameterized as

$$ SR={K}_r\times \sqrt{R_i^2+{R}_p^2+{R}_a^2+{R}_l^2} $$

where *K*_*r*_ is a factor of 1.2–1.5 that resulted from reconstruction methodologies [52], *R*_*i*_ is the intrinsic resolution given by *d*/2 with *d* as the crystal width, *R*_*p*_ is the error due to positron range (~ 0.2 mm for ^18^F), *R*_*a*_ is the error from PET non-collinearity given by 0.0022*D* with *D* as the ring diameter, and *R*_*l*_ as the decoding error due to anger logic positioning localization [31, 53]. The dPET system with 1:1 coupling of scintillator and SiPM detector does not need anger logic position decoding and therefore its degradation including distortions and edge effects is removed (*R*_*l*_ = 0). *K*_*r*_ is adjusted to be 1.43–1.54 for the dPET system after applying our NEMA spatial resolution results onto the above formula. A 10–20% improvement of the NEMA spatial resolution compared to the cPET [42] was obtained. In addition, depth-of-interaction (DOI) corrections may further improve the precision of annihilation localization and spatial resolution; it is expected that DOI integration will be available in future dPET systems.

**Fig. 11:**
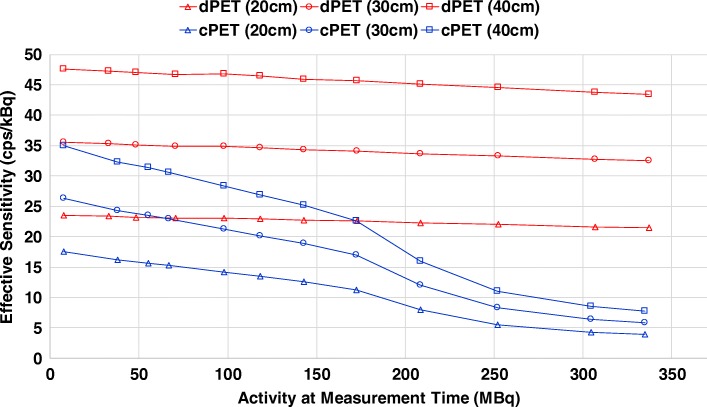
Effective sensitivity of dPET (322 ps) versus cPET (550 ps) as a function of ^18^F-FDG activities for object diameters of 20, 30, and 40 cm. It indicates a 1.3–5.5× effective sensitivity improvement of dPET with improved quality of counts compared to cPET over the measured activity of 7.4–337 MBq ^18^F-FDG, although the NEMA sensitivity of dPET is lower than cPET’s

NEMA sensitivity of the dPET system was found to be less than its former PMT PET/CT systems (Ingenuity TF 64, Gemini TF 64). In 3D mode, PET sensitivity degrades from peak at center to both edges in axial direction and is related to axial FOV length, PET overlap, detector ring size, and scintillator length. Typically, smaller detector rings with larger axial FOV lead to higher sensitivity scanners, and a PET overlap of 50% maintains a near uniform sensitivity at peak level. Compared to the prior cPET, the dPET system reduced the axial FOV length from 180 mm to 164 mm, the PET acquisition overlap from 53% to 39%, and the number of crystals by about 20% which in overall led to a reduced NEMA sensitivity from 7.3 (cPET) to 5.7 cps/kBq (dPET). The question to be asked is why reducing the system sensitivity by ~ 22% does not interfere with lesion detection but conversely has enabled improved image quality and lesion detectability of the dPET. This observation is consistent to the reduction of sensitivity resulting from a sparse-ring configuration [54]. From the sensitivity point of view, NEMA sensitivity measures only a system’s ability to convert photons to raw counts and does not take into account the quality of counts, and its reduction leads to fewer counts in quantity instead of quality. In another words, obtaining high-quality counts is more important than obtaining more lower-quality counts with a higher uncertainty, such as that the PSF resolution recovery curve of PMT systems tend to show long tails caused by ‘bad’ counts. For the dPET system, the SNR gain [55] from improved TOF timing resolution more than compensates for the somewhat reduced system sensitivity, and compared to non-TOF it translates NEMA sensitivity into an “effective sensitivity” improvement which contributes to ‘good’ counts instead of ‘more’ counts, as follows

$$ {S}_{\mathrm{eff}}(A)={S}_{\mathrm{NEMA}}(A)\times {G}_{\mathrm{TOF}}/{f}_d $$

$$ {G}_{\mathrm{TOF}}=\frac{D}{\Delta  x}=\frac{2D}{c\ \Delta  t} $$

where *S*_eff_ is the activity-dependent effective sensitivity, *S*_NEMA_ is the NEMA sensitivity, and *G*_TOF_ is the TOF gain defined as the object diameter (*D*) divided by the TOF localization uncertainty with *c* the speed of light and *∆t* the timing resolution, and *f*_*d*_ the dead time correction factor. The 322 ps dPET system presented an effective sensitivity with a gain of 4.1 for thin body size or brain PET (20 cm diameter), 6.2 for average body size (30 cm), and 8.3 for large body size (40 cm) acquisitions, which contribute to better images of high-quality counts, although the NEMA sensitivity is lower. An example of effective sensitivity comparing dPET with cPET is shown in Fig. 11. A potential advantage of the dPET system which may generate good image quality under low sensitivity

Image noise is usually characterized by NECR which is proportional to SNR and frequently used to compare the performance between different PET systems. In this study, even though it is not clear whether or not the measured maximum NECR of 171 kcps at 50.5 kBq/mL is the peak NECR, it seems that the plateau appears around this level. In addition, the dPET system with the introduced 1:1 coupling and vastly increased number of signal processing units allows it to handle far more counts per unit time than traditional PMT systems, which technically translates to a much higher peak true count rate. Despite using higher than usual ^18^F activity of ~ 1100 MBq for the NEMA count loss test, the testing failed to disclose the peak true count rate and its peak activity concentration of the dPET system. In our recent NEMA test using > 3000 MBq ^18^F (> 80 mCi, ongoing different project and not included in this work), the system peak true count rate appears to be close to 900 kcps at ~ 85 kBq/mL. This ultra-wide dynamic range of count rates can hopefully provide significant benefits to high count rates acquisitions such as cardiac perfusion PET.

Some factors may contribute to the appearance of artifacts on the cPET compared to the dPET at high SBRs in Fig. 8c. First of all, it is difficult to visually identify the existing artifacts and deformation on the phantom cPET images if using a wider SUV window to display, while they become apparently visible when the window-level settings are calibrated on similar background levels (e.g., 0–5 SUV), as shown in Fig. 8c. Secondly, the NEMA NEC body phantom has a volume of ~ 10 l with a filled activity of ~ 185 MBq (~ 5 mCi) ^18^F-FDG. This leads to an activity concentration of ~ 18.5 kBq/mL, which is high for the phantom particularly resulting in high count rate spheres when increasing SBRs. It asks for a system with larger dynamic range of count rate performance capability (such as NECR, the plateau @~ 50.5 kBq/mL for the dPET compared to the peak @15.7 kBq/mL for the cPET). Third, the timing resolution is very robust for the dPET (Fig. 7a) however has a big degradation for cPET system as increasing activity concentrations [42]. As a result, a substantial difference of effective NECR, which takes into consideration the TOF gain and the dead time correction factor, was found between both systems with a quick drop for the cPET (~ 50% drop of effective NECR from @15.7 kBq/mL to @25 kBq/mL) compared to a continued rise for the dPET (~ 55% increase from @15.7 kBq/mL to @~ 50.5 kBq/mL). This may cause the changes of count rate behavior and count quality at the ~ 18.5 kBq/mL activity concentration level.

While the broader clinical assessment is beyond the scope of this paper, we found in intra-individual comparison consistently improved image quality, detection, and classification of small lesions. It demonstrated that higher definition reconstruction appears to be of major clinical relevance for improved lesion detectability and characterization of lesion heterogeneity without increasing diagnostic ambiguity. It is expected that both the clinical sensitivity and specificity will be improved by dPET.

## Conclusions

This study evaluated system performance of a new generation, solid-state digital photon counting PET/CT representing the first such clinical system used for oncologic PET imaging. The system demonstrates excellent performance characteristics and stability over a 31-month monitoring period. Our findings reveal for the DPC PET clinical opportunities with improved image quality, lesion detectability and diagnostic confidence, and promising capabilities for performing faster and lower dose PET.

## Abbreviations

APD: Avalanche photodiode
cPET: Conventional PMT-based PET/CT
CQIE: Center for Quantitative Imaging Excellence
DCR: Dark count rate
DOI: Depth-of-interaction
DPC: Digital photon counting
dPET: DPC PET/CT
FWHM: Full width at half-maximum
FWTM: Full width at tenth-maximum
HD: High definition
LYSO: Lutetiumyttrium oxyorthosilicate
MOS: Metal oxide semiconductor
NECR: Noise equivalent count rate
NEMA: National Electrical Manufacturers Association
OSU: The Ohio State University
PMT: Photomultiplier tubes
PVE: Partial volume effect
QC: Quality check
RC: Recovery coefficient
SBR: Sphere-to-background ratio
SD: Standard definition
SiPM: Silicon photomultiplier
SPAD: Single photon avalanche diode
SUV: Standardized uptake value
TOF: Time of flight
UHD: Ultra-high definition
WCIBMI: Wright Center of Innovation in Biomedical Imaging
